# Experimentalismo Brabo, art, and culture in territories of exclusion: an interview with Leo Salo

**DOI:** 10.1590/S0104-59702023000100064

**Published:** 2023-11-10

**Authors:** Leonardo de Souza Melo, Elis Regina Barbosa Angelo

**Affiliations:** i Ativista social, provocador, Coletivo Experimentalismo Brabo. Rio de Janeiro – RJ – Brasil salorj@gmail.com; ii Professora, Departamento de Administração e Turismo e Programa de Pós-graduação em Patrimônio, Cultura e Sociedade/Universidade Federal Rural do Rio de Janeiro. Nova Iguaçu – RJ – Brasil elis@familiaangelo.com

**Keywords:** Territory, Manguinhos, Cordel literature, Clowning, Social projects

## Abstract

This interview with Leo Salo aims to understand how methods to foster interaction in excluded territories were created through the active listening of the community in actions developed as part of the Passeio Brabo project, designed to highlight the work undertaken in Rio de Janeiro’s Manguinhos community. With its clown parades, Experimentalismo Brabo endeavors to apprehend the realities of the community by listening to its demands inside its own territory, alleys, and favelas, reaching out to the homes and individuals in the community space to create environments for interaction, active listening, and collaboration.

## Resumo

A entrevista com Leo Salo busca compreender a criação dos instrumentos de interação no território, promovidos por ações de escuta da comunidade por meio do projeto Passeio Brabo, com o intuito de trazer os trabalhos desenvolvidos na comunidade de Manguinhos, no Rio de Janeiro. As ações do grupo Experimentalismo Brabo procuram, por meio dos cortejos de palhaçaria, apreender realidades da comunidade pela escuta de suas demandas, que ocorrem dentro do território, dos becos e das favelas, chegando às casas e às pessoas no espaço de favela, a fim de criar cenários de interação, escuta e colaboração.

> *Passeio Brabo é o primeiro passo metodológico da atuação do Coletivo Experimentalismo Brabo no território* (Salo, 8 fev. 2014).

O território, uma das categorias mais estudadas da geografia, é muito discutido, principalmente no que concerne à contextualização de cidades, urbanização e mesmo com relação ao vivido pelos sujeitos históricos (Lefèbvre, 1991). A interação humana é uma das vertentes do conceito de território amplamente debatido nas ciências humanas. Nessa vertente busca-se no espaço social vivido o âmago das interações e seus conflitos, especialmente as relações de poder, de apropriação e as redes de dominação (Harvey, 2005). Há também outros fatores relevantes que fortalecem as interações sociais e, de certo modo, suas ramificações entre os pares. Muitos debates acerca das relações intrinsecamente ligadas ao poder e à natureza como protagonistas desses territórios favorecem as relações humanas por meio de suas experiências cotidianas (Angelo, Fogaça, Barbosa, 2020; Angelo, 2021). As funcionalidades desses territórios também têm sido palcos privilegiados de debates na academia, trazendo vivências locais e suas dinâmicas, como é o caso de diversas publicações acerca dos projetos desenvolvidos no bairro de Manguinhos, no Rio de Janeiro.

Um dos trabalhos, *Histórias de pessoas e lugares: memórias das comunidades de Manguinhos*, de Tania Maria Fernandes e Renato Gama-Rosa Costa (2009), trata do contexto urbano complexo e vulnerável e envolve reflexões sobre as diversas favelas ao redor da Fundação Oswaldo Cruz (Fiocruz), com seus inúmeros problemas socioambientais e sanitários. Esse trabalho privilegia os desafios de pesquisadores da Fiocruz acerca das diversas realidades sobre a região, gerando projetos como o Laboratório Territorial de Manguinhos, desenvolvido na Fiocruz, uma parceria entre a Escola Nacional de Saúde Pública Sergio Arouca, a Escola Politécnica Joaquim Venâncio, a Casa de Oswaldo Cruz e o Instituto de Comunicação e Informação Científica e Tecnológica em Saúde (Icict). Nesse contexto, vêm se estabelecendo diálogos e atividades como uma proposta gratificante no desempenho de muitas pesquisas-ação. Os mais variados projetos se alimentam das reflexões e práticas sobre as relações dos sujeitos com o território, na sua multidimensionalidade, especialmente a partir das narrativas dos sujeitos e suas relações, favorecendo-lhes o entendimento e engajamento na promoção da saúde de suas comunidades.

Aspectos relevantes sobre o território de Manguinhos e a transformação nele produzida ao longo de sua criação e de seu estabelecimento estão num dos trabalhos que concatenam esforços sobre a trajetória do Instituto Oswaldo Cruz acerca das dinâmicas temporais que se entrecruzam na formação das práticas e saberes em distintos campos: "Retratos do cotidiano em Manguinhos", de Jaime Benchimol (2022), buscou reconstituir o cotidiano no instituto, trazendo expressivamente a relevância de Manguinhos como uma escola de formação e transformação do território, que inclui não somente gerações de cientistas, mas toda a sociedade.

Em meio aos diversos projetos elaborados para os desafios desse território, com suas dimensões e dinâmicas próprias, nasceu o Experimentalismo Brabo, produto de um trabalho coletivo que busca interação e ação de escuta e produção cordelística, privilegiando dimensionar os problemas individuais e coletivos dessas comunidades do território de Manguinhos.

Esse projeto foi idealizado por Catia Nascimento, Felipe Eugênio e Leonardo de Souza Melo, ou Leo Salo, bibliotecário por formação, palhaço, fotógrafo e cordelista, como se define. Leo Salo sempre refletiu sobre as relações entre arte/ciência, arte/cultura pelo ponto de vista da informação em saúde. Em seu mestrado em ciências pelo Instituto de Comunicação e Informação Científica e Tecnologia em Saúde, da Fiocruz, discutiu palhaçaria e terceira idade no âmbito de políticas de formação e comunicação em saúde na edição do espaço entre ciência e sociedade. Na ocasião, foi orientado pela professora Cícera Henrique da Silva. Em 2013, fundou, juntamente com Catia e Felipe, o projeto Experimentalismo Brabo, no complexo de favelas de Manguinhos. Desde então, o grupo realiza intervenções sobre cultura da paz, afeto, solidariedade e cooperativismo.

Nesse sentido, a proposta do grupo, intitulado coletivo, enfatiza como missão o propósito de provocação artística no intuito de refletir sobre afeto, solidariedade e cultura da paz. Para eles, o "Experimentalismo Brabo ... é a liberdade de arregaçar com a arte: desnudando cultura, esgarçando tabus, preconceitos, desigualdades sociais, identidades e… Reticências!" (Pela..., 14 dez. 2021). A ação se dá por meio do entretenimento proporcionado pela representação do palhaço, que, no percurso dos circuitos, faz a escuta dos moradores das favelas integrantes do território de Manguinhos, buscando, com práticas de arte e cultura em territórios de exclusão, observar os sujeitos locais como seres pensantes, como seres políticos, como seres sociais (Fontanetto, 6 ago. 2021).

A metodologia da tecnologia social desse projeto traz as práticas de ação em três momentos distintos, por meio de passos que são definidos a partir do que cada território oferece. Esses passos são: (1) escuta: que funciona como um diagnóstico de demandas e possibilidades de atuação; (2) ações: que se constituem após a escuta com finalidade específica de criação de brincadeiras e atividades artísticas para o território, a fim de trabalhar a cultura da paz, do afeto, da solidariedade e do cooperativismo; e (3) dar voz à cultura, memória e história de vida dos membros da comunidade.

Esse processo de audição ou escuta pode ser entendido como uma metodologia que busca compreender os significados das narrativas e também os silenciamentos nelas contidos. Após a escuta, a intervenção entra em cena como um processo interacionista, no qual os sujeitos se tornam mais receptivos e abertos para permitir ao oprimido dar voz a sua cultura, a sua memória e a sua história de vida.

Na intervenção, utilizam-se recursos lúdicos como música, brincadeiras (chamada pelo grupo de brincação), contação de histórias, poesia e palhaçaria, bem como tudo que permita brincar e dialogar com o território.

Outro aspecto desses percursos é a distribuição de cordéis, que também funcionam como formas de educar e interagir, pois são produzidos a partir de histórias e narrativas desse território. Nesses folhetos, as histórias das comunidades de Manguinhos são contadas por meio da poesia popular e registradas nos cordéis que serão distribuídos nas escolas, levando consigo as narrativas desses protagonistas e de seus territórios.

Muitos desses folhetos contam as histórias de moradores, militantes, ativistas e pessoas comuns com seus cotidianos de trabalho, luta e lideranças. Aspectos de suas vidas são incluídos nas histórias, também construídas por escuta da população.

Há que destacar a importância que a literatura de cordel conquistou nos últimos anos, a partir de seu reconhecimento e salvaguarda como Patrimônio Cultural do Brasil pelo Instituto do Patrimônio Histórico e Artístico Nacional (Iphan, 2018).

Essa conquista fortaleceu as práticas do saber-fazer dos protagonistas e detentores desse conhecimento popular, especialmente para "a gestão de seu patrimônio, a articulação interinstitucional em prol do bem registrado e a ampliação da sustentabilidade dos Patrimônios Culturais do Brasil" (Iphan, 2018).

O projeto Memória de Manguinhos em Cordel, de 2015, introduzido por Leo Salo, também protagonista desse saber-fazer, com o apoio da Awesome Foundation (fundação americana que financia uma ampla gama de projetos, incluindo artes, ciências e causas sociais) e do Museu da Vida da Casa de Oswaldo Cruz, resultou na publicação da série de cordéis que foi distribuída gratuitamente em escolas da região. O intuito desse projeto foi aproximar as pessoas do território relatando suas histórias de luta, trabalho e superação, além das manifestações artísticas e culturais que a própria comunidade acadêmica do *campus* Manguinhos muitas vezes desconhece.

O trajeto de Leo Salo justifica a importância de sua atuação e da atuação do coletivo na construção da cultura da paz, na sedimentação de um processo de memória, história e inclusão que fomenta ações para a saúde mental e social nesse território, ao dar voz a esses atores, na mesma medida em que colabora com a missão da Fundação Oswaldo Cruz na prevenção de patologias, inclusive sociais.

A ideia de criar o projeto Experimentalismo Brabo foi, segundo Leo Salo, arregaçar as mangas e trazer à comunidade e à sociedade em geral o envolvimento com a arte, principalmente evidenciar as desigualdades sociais e identitárias e, segundo ele, as reticências, ou seja, os silenciamentos.

Outros fatores de extrema relevância são compreendidos no Experimentalismo Brabo como referenciais para o acesso a essa produção de conhecimento sensível e cidadã, antenada às necessidades locais, e aqui entram as condições de moradia, transporte, poluição, enchentes, problemas de saúde, infraestrutura de apoio entre muitos outros aspectos que farão a diferença nas formas de assistencialismo não como um processo puramente filantrópico, mas de colaboração, mutualidade e parceria. A equipe do projeto é formada hoje por Leo Salo, Catia Nascimento, Diana Nunes, Karen Guimarães, Rafaele Reis e Wildson França.

Entre os diversos projetos, estão: Picnic Literário de Manguinhos, que tem o intuito de promover a leitura e a valorização da cultura local; Gotas de Brabeza, visando ouvir as condições vividas e experienciadas nas comunidades; Memórias de Manguinhos em cordel; Onde está a Alegria do Mundo?, que busca nas diferentes interpretações e concepções da alegria, situações, culturas e lugares para ações de intervenções de palhaçaria e contação de histórias, com o *slogan* "buscando a alegria do mundo", entre outros.

Nessa entrevista, entre outros objetivos, busca-se divulgar o grupo e suas ações de escuta, em especial, trazer o trabalho e o protagonismo de Leo Salo, atuante nas comunidades de Manguinhos como palhaço, cordelista, ouvinte e ativista social, privilegiando sua história para que a escuta possa conquistar outras dinâmicas do território em que se produzem tantos sentidos e silenciamentos. Ouvir é um caminho significativo na história da população e da sociedade carioca.

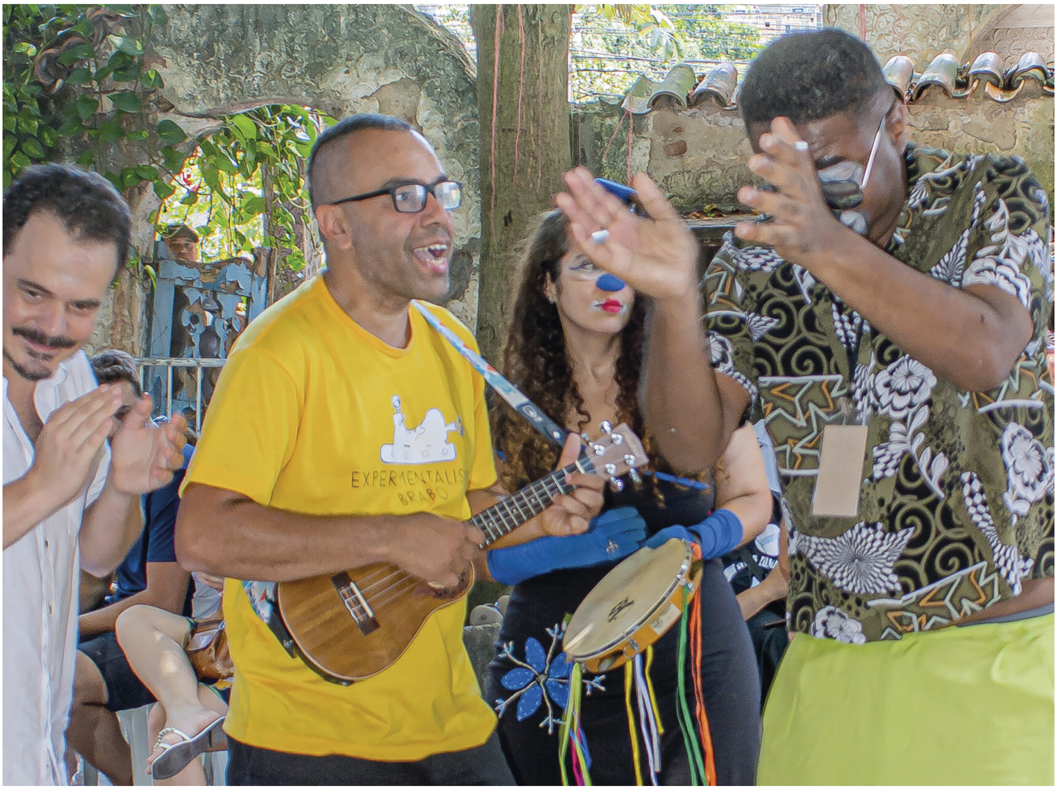

Figura 1: Apresentação de Leo Salo/Experimentalismo Brabo em Manguinhos, 2023 (acervo de Leo Salo)

*Elis: Como foi sua história com a literatura de cordel? Como começou e vem se transformando essa atividade na sua vida?*

Eu coordeno o coletivo cultural Experimentalismo Brabo. A ideia nasceu em 2013, em conversas que tive com a ativista Catia Nascimento e com o pesquisador em arte Felipe Eugênio. Após esses debates, aumentou nossa vontade de atuar com a provocação artística e definimos um foco: realizar intervenções sobre afeto, solidariedade e cultura da paz em territórios de exclusão. Convidamos então os artistas Wallace de Oliveira, Cléo Lima, Karen Guimarães e Drico Lima para integrar o coletivo, que nasce e realiza suas primeiras intervenções em Manguinhos. De início, fizemos cortejos de palhaços na favela, os quais chamamos de Passeios Brabos. Neles, a interação e a escuta nos becos e vielas nos levou a perceber que a visão de que a favela "não tem nada de bom" não existe apenas na cabeça de quem "é de fora", mas também estava presente no discurso de quem "é de dentro", ou seja, quem mora ou trabalha na favela. Dessa forma, buscamos uma ferramenta para que pudéssemos falar sobre atores e manifestações culturais relevantes do território em uma linguagem de fácil compreensão, mas que despertasse o interesse tanto de quem é quanto de quem não é de Manguinhos.

Depois de pensar e avaliar formatos, chegamos na proposta de trabalhar com a literatura de cordel. Então comecei a estudar esse estilo literário, buscando inspiração e orientação no contato com alguns poetas da Feira de Caruaru (PE). Em seguida procurei a Editora Coqueiro, em Recife (PE). Lá conheci o poeta pesquisador Meca Moreno e a editora Aninha Ferraz. Após essa imersão, comecei a escrever os primeiros folhetos. Desde então, posso dizer que o cordel definitivamente me pegou. E quando o cordel pega, ele não larga, não...

De início, escolhemos trabalhar no coletivo com a figura do palhaço, levando em conta a possibilidade de troca, e a escuta natural que o palhaço desenvolve em sua *performance*. Entendemos que seria bastante provocador utilizar essa perspectiva do palhaço para o diálogo com as pessoas do território. Começamos, então, a fazer os cortejos de palhaçaria, percorrendo os espaços, indo nas casas, chegando a pessoas que não estavam muito acostumadas a ter esse tipo de ação dentro do espaço de favela.

Normalmente, as ações artísticas e culturais acontecem em pontos específicos, uma praça, uma quadra, um campo, um botequim. Mas a gente queria ir até onde ninguém vai, e foi quando decidimos que, em vez de utilizar um ponto específico, a gente iria naqueles becos onde as intervenções artísticas não chegam. Nós chamamos esse cortejo de Passeio Brabo, justamente por isso, porque a gente vai onde tiver de ir e escuta quem tiver de escutar.

E dessa escuta a gente percebeu, como te disse, a necessidade de falar sobre memória: registrar e divulgar informação sobre os agentes e as manifestações artísticas e culturais importantes para Manguinhos. Elaboramos então o projeto Memória de Manguinhos em Cordel, que conta hoje com dez títulos publicados.

Independentemente da demanda que surgiu com o coletivo, eu, particularmente, já lia muito cordel, então esse tipo de literatura já estava presente na minha vida. No entanto, ao começar a escrever para esse fim, o cordel passou a ocupar um espaço bem maior na minha vida. Escrevi sobre outros temas, pesquisei sobre a história e as diferentes formas possíveis de apresentá-la e até ministrei algumas oficinas.

*Elis: Como a comunidade de Manguinhos apoia essa ideia? Como é que as pessoas, de formas diferentes, acabam abraçando o projeto? Nas casas, os membros das famílias, por faixas etárias, grupos ou indivíduos?*

Não sou "cria", mas sou visto como "criado" pela maior parte das pessoas que conhecem meu trabalho. Sou tido como alguém que, mesmo sendo de fora, já é de dentro do território.

Isso está diretamente ligado à boa receptividade que tive desde meus trabalhos pelo Icict/Fiocruz, e atualmente pelos trabalhos com o Experimentalismo Brabo, seja nosso cortejo de palhaços ou nossa poesia de cordel, eu pessoalmente sempre percebi uma recepção muito positiva.

O palhaço, por exemplo, deu um resultado incrível! Não foram apenas as crianças que interagiram; tivemos uma resposta muito boa também por parte dos adultos. Muitos viam no palhaço uma possibilidade de desabafar sobre seus problemas pessoais, e muitas vezes sobre questões de foro muito íntimo e pessoal. Alguns contavam sobre a dificuldade para agendar consultas ou procedimentos médicos, outros falavam sobre as suas doenças, problemas familiares e até mesmo sobre suas dores e segredos de amor. O palhaço conseguiu atingir todas as idades.

Com nossos folhetos de cordel, não foi diferente. Com uma linguagem simples, eles se tornaram outra forma de diálogo. Mas, dessa vez, um diálogo específico sobre memória, arte e cultura. Por opção, a maioria dos folhetos tem oito páginas. Tentamos publicar folhetos curtos para facilitar a leitura. Isso me dificulta demais na escrita, pois narrar os pontos interessantes da vida de uma pessoa em 24 estrofes é bastante trabalhoso. Mas a minha intenção é que a pessoa pegue aquele folheto e leia até o fim. Mesmo quem não tem o hábito da leitura.

Os nossos homenageados têm a sensação do que chamamos flores em vida, ou seja, de poder ver o reconhecimento da importância de suas ações para o território de Manguinhos. Eles e elas passam então a ser também divulgadores das publicações.

*Elis: E, nessas caminhadas, você acabou se deparando com outros cordelistas? Ou isso acabou unindo outros cordelistas desse território? Isso foi de certa forma uma chamada?*

Já escrevendo a série Memória de Manguinhos em Cordel, conheci a obra do cordelista Victor Lobisomem. Desde então, ele também passou a ser uma de minhas referências no cordel. Seu trabalho é magnífico, valorizando a cultura carioca, escrevendo sobre nomes como Paulo da Portela, dona Ivone Lara, sobre o Cacique de Ramos, entre outros. Posteriormente, conheci a pesquisa e o trabalho do paraibano Aderaldo Luciano. Com ele aprendi muito sobre a história do cordel. Todavia, devo dizer que não conheço outros cordelistas de Manguinhos especificamente.

*Elis: Existe um local de encontro? De produção? Disseminação?*

Nossos cordéis são distribuídos gratuitamente, graças ao apoio de nossos parceiros: Sindicato dos Servidores de Ciência, Tecnologia, Produção e Inovação em Saúde Pública (Asfoc-SN), o Museu da Vida e a Awesome Foundation. Mais recentemente, conseguimos imprimir folhetos também graças a editais da Lei Aldir Blanc. A distribuição geralmente acontece em algum evento cultural já existente, ou em eventos produzidos por nós, como o Sarau Afeto Brabo e o Piquenique Literário Experimenta.

*Elis: O que você acha que mudou para os cordelistas e mesmo para a literatura de cordel com o reconhecimento pelo Iphan como patrimônio cultural imaterial brasileiro?*

Cordel é um estilo literário brasileiro. E, como toda arte nascida aqui, é tida por muitos como uma "arte menor". É assim com várias outras manifestações artísticas e culturais nacionais, infelizmente. Esse reconhecimento do Iphan é muito importante. Dá visibilidade. É um primeiro passo, mas ainda há muito a ser feito! Apesar da visibilidade, eu lhe digo, por exemplo, que já vi instituição séria e grande promover oficina de cordel sem a presença de um cordelista ou de um pesquisador dessa literatura. Apesar de a instituição reconhecer a importância do cordel, esse é um exemplo de que nem sempre o reconhecimento e a vontade de falar sobre o tema estão atrelados ao cuidado de verificar que o cordel é um sistema literário e, como tal, identificar algum membro ativo dessa cadeia (escritor, editor, pesquisador) para a realização de eventuais atividades.

*Elis: A internet veio para ajudar ou é prejudicial à literatura de cordel?*

Veio para ajudar e ajudar muito. Por vezes uma pessoa interessada no cordel não encontra pares em sua região, e hoje ela pode facilmente interagir com cordelistas e pesquisadores em todo o Brasil. Eu mesmo já ministrei três oficinas virtuais, com participantes de vários estados do Brasil e participantes de fora do país.

*Elis: Ao todo, quantos cordéis você já escreveu. Quais os temas que já desenvolveu?*

Escrevi em torno de vinte cordéis, quase sempre, biográficos. Além da série sobre Manguinhos, tenho com o Experimentalismo Brabo uma série sobre artistas de Niterói. Em carreira solo, tenho algumas histórias de ficção e biografias sobre artistas negros. Além disso, integro hoje, juntamente com o guitarrista Michel Ramos e o dançarino Iguinho Imperador, o Fanquitoide, um projeto literomusical com histórias ficcionais sobre personagens suburbanos e/ou periféricos, cuja base literária é toda em cordel.

*Elis: Você acha importante o cordel nas escolas? Como isso ocorre em Manguinhos?*

Importante demais. É uma boa ferramenta de sedução para que os alunos possam conhecer mais o seu território e discutir de forma mais leve os conteúdos que a escola pode trabalhar em termos de reflexão sobre o local.

Em Manguinhos, eu destaco a Escola de Jovens e Adultos (EJA) da Escola Politécnica de Saúde Joaquim Venâncio, que utiliza bastante nossos cordéis. Também devo citar a Escola Municipal Professora Maria de Cerqueira e Silva e a Escola Municipal de Ensino Fundamental Dom João IV, que, respectivamente, com os professores Marco Aurélio e Anamô, fazem um belíssimo trabalho.

*Elis: Como vocês usam a ilustração? Quem faz a xilogravura?*

Fazemos sempre que possível. Carlos Henrique Soares e Tiago Carva foram os dois artistas que mais ilustraram capas.

*Elis: Como você divulga seu trabalho? E os outros cordelistas que conhece?*

Em Manguinhos, os principais canais de divulgação dos nossos cordéis são as escolas e os eventos que organizamos ou de que participamos no território. Os canais virtuais, onde os cordéis podem ser baixados, são o nosso *blog* (www.wordpress.com/ebrabo) e o Repositório Institucional da Fundação Oswaldo Cruz (Arca).

Cada poeta tem sua própria estratégia, que pode, por exemplo, passar pela divulgação em grupos de Facebook/Telegram/WhatsApp e pela participação em eventos culturais literários ou de arte em geral.

Mas é muito importante dizer que o movimento de um poeta divulgar o trabalho do outro faz alimentar a roda do “nós por nós”. Sabemos que nossa arte é tida por muitos como uma “arte menor”, e o trabalho que fazemos de divulgação sem competição do trabalho de nossos colegas faz a roda girar no sentido da valorização dessa arte que abraçamos.

*Elis: Sobre projetos e cursos nas áreas de saúde, arte e cultura em territórios favelizados, trabalhos tão relevantes para a história local, gostaria de ouvir um pouco mais.*

Conheci Manguinhos em 2013, como bolsista do Icict/Fiocruz. Aceitei na época um convite da pesquisadora Cristina Guimarães para coordenar com ela um projeto chamado Cais (Curso de Agentes Culturais pela Informação em Saúde), que pensava justamente essa relação entre saúde, arte e cultura, com vistas a fornecer subsídios informacionais para outras ações da própria fundação, além de fortalecer as iniciativas culturais locais. Pensar essa relação significa pensar a vida, a saúde, sobretudo a saúde mental num território tão estigmatizado pela ausência, em todas as esferas governamentais. Pensar essa relação é pensar o fortalecimento de boa parte das dinâmicas sociais desse território. Muitos setores da Fiocruz já perceberam isso. A Cooperação Social também se faz bastante presente, assim como o EJA da Escola Politécnica de Saúde Joaquim Venâncio e o Museu da Vida da Casa de Oswaldo Cruz, com as suas ações territorializadas.

Cabe, porém, destacar que não apenas ações vinculadas ao poder público conseguem abranger essa relação. O "Projeto Marias: como posso ajudar meu filho especial" – por exemplo, foi fundado, em Manguinhos, pela psicopedagoga Norma Maria, no intuito de fomentar a disseminação de informação no cuidado e nas garantias dos direitos das pessoas com deficiência, e, em suas ações, acaba trabalhando no escopo das relações entre arte, saúde e cultura na favela.

*Elis: Comente pontos que achar relevantes para essa divulgação da arte cordelista e da formação do cordel em Manguinhos.*

Manguinhos tem uma tradição poética muito forte. O trabalho específico com o cordel é uma ação do Experimentalismo Brabo, que publica meus folhetos. Todavia, se falarmos em poesia de modo geral, é importante salientar isso. A poesia em Manguinhos é entretenimento, expressão criativa, (re)conhecimento do território, mas também é uma ferramenta de transformação social. Acho interessante novamente destacar que, no caso dos cordéis da coleção Memórias de Manguinhos em Cordel, trata-se de uma ferramenta para registro e difusão da memória cultural local.

Há muitos anos acontece, por exemplo, o Sarau Poético de Manguinhos, coordenado pela Maura Santiago, que também foi homenageada em nossa série de cordéis. O sarau nasce no âmbito de um pré-vestibular comunitário, onde os professores buscavam um meio de tornar mais atrativa a discussão sobre a redação. A própria Maura já organizou também o Sarau Feminista e o Sarau Itinerante, por exemplo, além de vários outros.

Mais recentemente, por exemplo, temos a Roda Cultural do Pac'Stão, com XandyMC e seus amigos. Temos ainda o Slam Manguinhos, organizado com a Franciele Campos, Sergi Oliveira e uma turma muito potente. A Comissão de Preservação da Igreja de São Daniel Profeta (coordenada por Éric Gallo) também realizou recentemente uma oficina de poesia para falar sobre a importância da Igreja, que contou com a participação da cordelista Kaká Freitas.

Temos ainda muita gente fazendo poesia em Manguinhos, como Celeste Estrela, Maria do Socorro, Hugo Abreu, Leonício, Antonio Batista, Eliona Araújo, Elesbão Ribeiro (atuante no Sarau Poético de Manguinhos), Caio Oliveira (que foi meu aluno numa oficina de cordel) e muitos outros.

**Veja a relação de títulos publicados pelo projeto Memória de Manguinhos em Cordel**

Volume 1 – *Norma Maria*. Nascida e criada nas favelas de Manguinhos, a psicopedagoga Norma Maria idealizou e hoje coordena o "Projeto Marias: como posso ajudar meu filho especial".

Volume 2 – *Tia Lauzinha*. O Boteco da Lauzinha já é grande tradição e há mais de vinte anos na favela é atração. Quem conhece se encanta, guarda o bar no coração!

Volume 3 – *Viviellen*. O folheto mistura ficção e realidade, ao relacionar a história da personagem Viviellen com a memória de parte das manifestações culturais do território.

Volume 4 – *O castelo da Fiocruz*. A história da majestosa construção que se localiza em Manguinhos.

Volume 5 – *Celeste Estrela*. Começou a fazer arte antes de adolescente. Criativa desde cedo, querida por toda a gente… Ela é Celeste Estrela: poetisa competente!

Volume 6 – *Iguinho Imperador*. Rei na dança em Manguinhos. Ouve essa, morador: nome bem reconhecido, da arte um batalhador! Hoje trago sua história, em versos de cantador…

Volume 7 – *Geraldo de Andrade*. Seu Geraldo e o teatro: uma vida de amor. É na arte seu espaço de protesto e de clamor: hoje fala abertamente, não precisa mais temor!

Volume 8 – *O Padre da Pá Virada*. Quem conhecer o Gegê pode não acreditar, é Padre da Pá Virada, pra com fé e amor lutar: junto da nossa favela, liderança exercitar.

Volume 9 – *As minas da bola*. Garotas do Mandela são estrelas a brilhar: tendo chance, elas mostram como sabem trabalhar. Querem oportunidade, caminho para trilhar.

Volume 10 – *Maura Santiago*. Maura nasceu em Manguinhos, milita bem na cultura. Tem grande protagonismo, valorizando a leitura!

Volume 11 – *Daiana e o balé em Manguinhos*. "Dançando pra não dançar", projeto reconhecido: teve aluna do Mandela com talento garantido, que propôs um "corre" bom. Igualmente conhecido... (Salo, 2022).
